# Sertraline-Related Bleeding Tendency: Could It Be Dose-Dependent?

**Published:** 2014

**Authors:** Mahin Eslami Shahrbabki, Amir Eslami Shahrbabaki

**Affiliations:** 1 Assistant Professor, Child and Adolescent Psychiatrist, Neuroscience Research Center, Institue of Neuropharmacology AND Department of Psychiatry, Afzalipour School of Medicine, Shahid Beheshti Hospital, Kerman University of Medical Sciences, Kerman, Iran.; 2 Researcher, Department of Sports Medicine, School of Medicine, Tehran University of Medical Sciences, Tehran, Iran.

**Keywords:** Bleeding Tendency, Dose-Dependent, Selective Serotonin Reuptake Inhibitors

## Abstract

Selective serotonin reuptake inhibitors (SSRIs) are reported to be associated with increased bleeding tendency. While findings of recent studies explain a lot about the pathophysiology of this side-effect, there is a general tendency to discontinue SSRIs as harmful medications. We report two instances of dose-dependent relations between sertraline and bleeding tendency. Bleeding diathesis was alleviated by adjusting dosage of medication. It could be argued that benefits of SSRIs could outweigh this potential and probably avoidable side-effect; if dose-adjustment is properly implemented.

## Introduction

Increased bleeding tendency is a relatively infrequent adverse effect of selective serotonin reuptake inhibitors (SSRIs). Although they are used extensively patients with psychiatric problems, occurrence of this side-effect is clinically important. Several mechanisms have been suggested for bleeding induced by SSRIs: (i) serotonin uptake from blood into platelet is inhibited leading to decreasing serotonin stores in platelets which affects platelet aggregation, (ii) increasing gastric acid secretion might induce gastrointestinal (GI) bleeding; (iii) concomitantly use of SSRIs with non-steroidal anti-inflammatory drugs or anti-platelet drugs can increase the risk of bleeding (1).

Among SSRIs, those with the higher degree of serotonin reuptake inhibition, fluoxetine, paroxetine, and sertraline are more frequently associated with abnormal bleeding (2). SSRI use is associated with doubled risk of upper GI bleeding; bleeding at other sites has been less commonly reported (1). The relationship of this adverse effect with a dose of the drug has not been appreciated enough as clinicians are used to discontinue the drug immediately after this side-effect happened (3-8). Here, two cases of dose-dependent bleeding induced by sertraline are reported.

## Case Report

The first patient was an 11-year-old boy with a history of separation anxiety disorder, who was started on sertraline with the diagnosis of obsessive-compulsive disorder (OCD). The dose of the medication had been gradually increased to 75 mg twice a day during a 2-week period, when he complained of massive epistaxis. Initial workup including clinical assessments and laboratory tests was performed, which was inconclusive. Medication dose was reduced to 50 mg twice a day and bleeding ceased. After about 5 days the dosage was increased again and then a more severe epistaxis episode was reported by his family after just one day of the new dosage. Patient was referred for an otorhinolaryngology consultation, and subsequently underwent heat-coagulation and tamponing. Still, after removing the tampon epistaxis continued. Eventually, the dose of sertraline was reduced, and bleeding ceased without relapsing. Sertraline was not changed to another antidepressant because the patient was satisfied with the medication.

The second patient was a 36-year-old woman who was started on sertraline with a diagnosis of severe generalized anxiety disorder and OCD, complicated by signs of depression. During 8 weeks the dosage was gradually increased to 100 mg twice a day. After achieving the maximum dose, she presented with microscopic hematuria detected by a urine analysis, taking for another reason. When the dosage of the medication was reduced to 75 mg twice a day, hematuria resolved. Then, after the previous dosage was resumed, hematuria recurred. Finally, the dosage was maintained at 75 mg, twice a day without any hematuria.

## Discussion

SSRIs, with their particular side-effects, are widely used. Their relative safety compared to other antidepressants, and their broad implications in psychiatric disorders make them a favorite choice for many clinicians (3). Their side-effects, such as increased bleeding tendency, are not new phenomena and have been reported by other practitioners (3-9). Hemostasis happens as an orchestrated cascade of four main events: platelet plug formation or primary hemostasis, clot formation, antithrombotic activation and fibrinolysis (2). Abnormalities of the first and second steps of this cascade have been reported in patients treated with antidepressants due to alteration in the metabolism of 5-hydroxytriptamine (serotonin) (2). Various reasons for the phenomenon have been suggested, but according to the findings of Halperin and Reber (2) alterations most commonly occur in the first phase of hemostasis, that is plug formation, and a decrease in aggregation and activity of platelets could be observed. This latter feature stems from the fact that serotonin is secreted by platelets and plays a role in platelet aggregation (2). Notably, pre-existing disorders in platelet functions, such as modified response of platelets to serotonergic stimuli, may contribute to bleeding diathesis in some patients treated with antidepressants (10).

The relationship of this side-effect with the dosage of the drug has not been appreciated up to now. Although previous studies have reported stopping SSRIs immediately after bleeding adverse reaction, we suggest dose reduction in this situation. It should be mentioned that dense granules in platelets contain serotonin (2, 11, 12) and this side-effect is probably through serotonin reuptake inhibitor mechanism in platelet. Therefore, it might be dose-dependent like other side-effects with a similar mechanism. We hypothesized that if this association is dose-dependent; there might be a threshold before symptoms appear. Thus, resolution and commencing of the symptoms around a specific dosage of the medication suggested a possibility for this association. Moreover, individual differences such as different levels of activity for cytochrome systems exist. This article is a case report with a number of uncontrolled factors; the observation bias should be considered. Large randomized controlled trials are required to answer the questions we raised here. In conclusion, we suggest that each case of bleeding tendency after prescribing SSRIs should be handled individually.

## Conclusion

Bleeding diathesis is a well-documented side-effect of SSRIs with varying management strategies. Our findings suggested that dose-adjustment could alleviate patients’ symptoms and each patient should be handled individually. 

## Authors' contributions

MESh and AESh reviewed the scientific literature and prepared the manuscript. MESh participated in the acquisition and interpretation of laboratory and clinical data. Both authors contributed to the final version, carried out the clinical case report, read and approved the final manuscript.

## Notes

**Declaration of interest:** None.
